# Combining radiation with PI3K isoform-selective inhibitor administration increases radiosensitivity and suppresses tumor growth in non-small cell lung cancer

**DOI:** 10.1093/jrr/rrac018

**Published:** 2022-05-09

**Authors:** Mi Youn Seol, Seo Hee Choi, Hong In Yoon

**Affiliations:** Department of Radiation Oncology, Yonsei Cancer Center, Yonsei University College of Medicine, Seoul 03722, Republic of Korea; Department of Radiation Oncology, Yongin Severance Hospital, Yonsei University College of Medicine, Yongin, Gyeonggi-do, 16995, Republic of Korea; Department of Radiation Oncology, Yonsei Cancer Center, Yonsei University College of Medicine, Seoul 03722, Republic of Korea

**Keywords:** radioresistance, PI3K isoform, radiation, radiosensitivity, non-small cell lung cancer (NSCLC)

## Abstract

Non-small cell lung cancer (NSCLC) is a malignant lung tumor with a dismal prognosis. The activation of the phosphoinositide 3-kinase (PI3K)/AKT signaling pathway is common in many tumor types including NSCLC, which results in radioresistance and changes in the tumor microenvironment. Although pan-PI3K inhibitors have been tested in clinical trials to overcome radioresistance, concerns regarding their excessive side effects led to the consideration of selective inhibition of PI3K isoforms. In this study, we assessed whether combining radiation with the administration of the PI3K isoform-selective inhibitors reduces radioresistance and tumor growth in NSCLC. Inhibition of the PI3K/AKT pathway enhanced radiosensitivity substantially, and PI3K-α inhibitor showed superior radiosensitizing effect similar to PI3K pan-inhibitor, both *in vitro* and *in vivo*. Additionally, a significant increase in DNA double-strand breaks (DSB) and a decrease in migration ability were observed. Our study revealed that combining radiation and the PI3K-α isoform improved radiosensitivity that resulted in a significant delay in tumor growth and improved survival rate.

## INTRODUCTION

Non-small cell lung cancer (NSCLC) is a major cause of cancer death [1]. NSCLC accounts for 80–85% of all lung cancer cases. Despite the advancement in anti-cancer therapy and drug development, the estimated 5-year overall survival rate is only 16% [2]. The current treatment options for NSCLC, which include surgical resection, radiotherapy and systemic chemotherapy, are determined based on the severity of the disease. Radiotherapy is an important treatment option for non-metastatic as well as metastatic disease. However, radioresistance which might increase as the radiation dose increases or when the re-irradiation is performed remains a significant therapeutic obstacle. Radioresistance has been shown to contribute to cancer therapy failure, recurrence and metastasis [3].

The phosphatidylinositol-3-kinase (PI3K)/protein kinase B (AKT) signaling pathway has been identified as one of the critical signaling pathways involved in cancer cell growth, survival and proliferation. Furthermore, this pathway is also known to be involved in the mechanisms of tumor-specific radioresistance [4–7]. Inhibition of the PI3K/AKT signaling pathway can increase the efficacy of radiotherapy in several different ways. Firstly, radiation-induced DNA double-strands breaks (DSB) induces phosphorylation of AKT both at threonine 308 and serine 473 and activates downstream signaling [8]. To improve the radiosensitivity, increased activation of the PI3K/AKT signaling pathway should be suppressed [9–11]. Secondly, inhibition of PI3K/AKT signaling can sensitize cancer cells to radiation-induced cytotoxicity [12–14]. And lastly, several recent studies have demonstrated that epithelial-mesenchymal transition (EMT) is associated with radioresistance of NSCLCs [15–17]. EMT plays an important role in the regulation of tumor aggressiveness and is associated with an unfavorable prognosis [18]. PI3K/AKT signaling pathway is known to play an important role in this EMT process [4, 19].

There is growing evidence supporting the use of PI3K inhibitors as radiosensitizers. However, the pan-PI3K inhibitor that suppresses all PI3K isoforms can cause side effects such as hyperglycemia, rash, neutropenia, diarrhea, neuropsychiatric effects, nausea and fatigue [20]. The use of inhibitors selective toward different PI3K isoforms (p110α, p110β, p110γ and p110δ) can be an effective alternative for regulating radioresistance. It is expected that selective inhibition of PI3K isoforms will cause a reduction in side effects while reducing radioresistance when combined with radiation. Recently, PI3K isoform-specific inhibitors have been developed and are being studied in combination with or without radiotherapy in clinical trials [4, 21].

In this study, we hypothesized that the combination of radiation and PI3K isoform inhibitors will increase radiosensitivity and suppress tumor development in NSCLC. The goal of this study was to develop a new radiation-PI3K isoform-selective inhibitor combination therapy to overcome radioresistance and improve the response rate of tumors to the radio therapy.

## MATERIAL & METHOD

### Cell line and cell culture

LLC1 (mouse lung carcinoma cell line) and A549 (human lung carcinoma cell line) were used in this study. Both LLC1 and A549 cells were plated in Dulbecco Modified Eagle Medium containing 10% fetal bovine serum (FBS) and 1% penicillin-streptomycin (P/S) and then cultured at 37°C in a 5% CO_2,_ humidified environment.

### Radiation

In the *in vitro* experiment, NSCLC cell lines were irradiated with a single dose of 2 Gy using X-rad 320 (Precision X-Ray, North Branford, CT, USA) for colony formation assay. 4 Gy and 6 Gy irradiation was applied with two repeated doses and three repeated doses of 2 Gy, respectively. Radiation fraction was operated at 320 kVp and 12.5 mA with 2.0 mm Al filtration and a dose rate of 4.76 cGy/sec.

### PI3K isoform-selective inhibitors

Four isoform-selective PI3K inhibitors were used in this study. Pictilisib (GDC-0941) is a potent inhibitor of PI3Kα/δ with modest selectivity against p110β (11-fold) and p110γ (25-fold). Taselisib (GDC-0032) is a PI3K inhibitor with potent inhibitory activity against p110α(PI3K-α inhibitor). Idelalisib (CAL-101, GS-1101) is a p110δ selective inhibitor (PI3K- δ inhibitor). Duvelisib (IPI-145) is a p100δ selective inhibitor that inhibits p110δ, P110γ, p110β and p110α(PI3K- γ/δ inhibitor). All inhibitors were purchased from Selleckchem (Houston, USA).

### Western blotting

The markers of the PI3K-AKT signaling pathway, phosphorylated AKT and double-stranded DNA break marker γ-H2AX were analyzed using Western blot. Protein was extracted from whole-cell lysates using a Cell Extraction Buffer (FNN0011, Thermo Fisher, Waltham, Massachusetts, USA) containing Halt™ Protease and Phosphatase Inhibitor Cocktail (78 440, Thermo Fisher, Waltham, Massachusetts, USA). Protein concentration was measured by BCA protein assay Kit (23 227, Thermo Fisher, Waltham, Massachusetts, USA). For Western blot analysis, protein extracts were separated via sodium dodecyl sulfate-polyacrylamide gel electrophoresis and transferred onto polyvinylidene difluoride membranes. The membranes were blocked with 3% bovine serum albumin (BSA) in tris-buffered saline with 0.1% Tween 20 (TBST) for 1 hour. The membranes were incubated overnight at 4°C in primary antibodies and washed three times for 10 minutes with TBST. Next, the blots were incubated in the horseradish peroxidase (HRP)-conjugated secondary antibody for 1 hour at room temperature and washed with TBST three times for 10 minutes. After the last wash, the membranes were incubated in enhanced chemiluminescence (ECL) solution (WESTSAVE ECL Solution, AbFrontier, Seoul, Republic of Korea) and exposed to X-ray films. The detailed information on antibodies used in Western blotting is provided in Supplementary Table 1.

### MTT assay

The effect of the PI3K-isoform inhibitors on cell proliferation was measured using the 3-(4,5-dimethylthiazol-2-yl)-2,5-diphenyltetrazolium bromide (MTT; Sigma-Aldrich, St. Louis, MO, USA) colorimetric assay. Approximately 1000–2000 cells per well were seeded on 96-well plates with 100 μL of DMEM growth medium and incubated overnight in a 37°C CO2 incubator. Then, 10% MTT solution in growth medium was added to each well. The plate was incubated in a 37°C CO2 incubator for 4 h. Thereafter, formazan crystals were dissolved in 100 μL of DMSO. Absorbance was measured at 590 nm using a microplate reader (VERSA max, Molecular Devices, San Jose, California, USA).

### γ-H2AX focus formation

After the irradiation of 2 Gy using X-RAD 320 (Precision X-Ray, North Branford, CT, USA), 2000 cells were plated on 4-well slide plates. After plating, the cells were treated with PI3K kinase inhibitors (GDC0941, GDC0032, CAL101 and IPI145) for 12 hours. The cells were fixed by a 4% paraformaldehyde solution. The cells were permeabilized by incubating in phosphate-buffered saline (PBS) containing 0.25% Triton X-100 for 10 min followed by washing three times with PBS for 5 min. Next, the cells were blocked with 1% BSA in PBS with 0.1% Tween 20 (PBST) for 1 h. The cells were incubated overnight at 4°C with anti-phospho-γ-H2AX antibody (05636I, Millipore, Burlington, Massachusetts, USA), and washed three times for 5 minutes with PBST. The cells were incubated with a fluorescein isothiocyanate-labeled secondary antibody for 1 h in the dark and mounted with VECTASHIELD solution (94 010, Vector Laboratories, Burlingame USA). Observation phospho-γ-H2AX foci were observed and fluorescence images were acquired using the Zeiss LSM 700 confocal fluorescence microscope.

### Colony formation assay

The cells irradiated using X-RAD 320 (Precision X-Ray, North Branford, CT, USA) were plated in 6-well plates in triplicate. After plating, the cells were treated with PI3K kinase inhibitors (GDC0941, GDC0032, CAL101 and IPI145). The cells were incubated for 9–12 days at 37°C in a 5% CO_2_ and humidified environment. Colonies were counted by staining the cells with 0.5% crystal violet.

### Scratch-wound healing assay

To measure the cell migration, A549 cell lines were seeded into 6-well plates containing RPMI media with 10% FBS, 1% P/S and cultured to approximately 100% confluence. Next, the cell monolayers were denuded at the median axes with sterile 1 mL pipette tips. The scratch-wound healing by cell migration was monitored over time, and images were captured using a light microscope with a 10× objective lens at the 0 and 48 h time-points after the denudation.

### *In vivo* combination therapy of mouse xenograft model

The mouse xenograft model was established in the C57BL/6 strain. LLC1 cells (1 × 10^6^ cells in 100 μL Dulbecco’s phosphate buffered saline) were injected subcutaneously into the mouse thigh. When tumor size reached approximately 100 mm^3^, the mice were randomly divided into four groups: vehicle control, radiation, GDC0032-treated and GDC0032+radiation-treated. GDC0032 was formulated in DMSO and intraperitoneally injected at a dose of 5 mg/kg one h after the irradiation. Mice in RT groups were irradiated with a single dose of 5 Gy fractions one time.

### Statistical analysis

The Student’s t-test was used to assess the difference in continuous variables of two groups. A repeated-measures ANOVA was used to compare changes in the tumor volume in each treatment group which was measured at eight different time-points (6, 8, 11, 14, 17, 20, 23 and 27 days). All the experiments were repeated at least three times. All the data were presented as the mean ± standard deviation. Data were plotted using GraphPad Prism 5.0 (GraphPad Software Inc, California, USA). Statistical analyses were performed using SPSS software, version 25.0 (SPSS Inc, Chicago, Illinois). A 2-sided *P*-value <0.05 was considered significant (*P* < 0.05 (^*^), *P* < 0.01 (^*^^*^) and *P* < 0.001(^*^^*^^*^)).

## RESULTS

### AKT phosphorylation in LLC1 tumor tissue

**Fig. 1:**
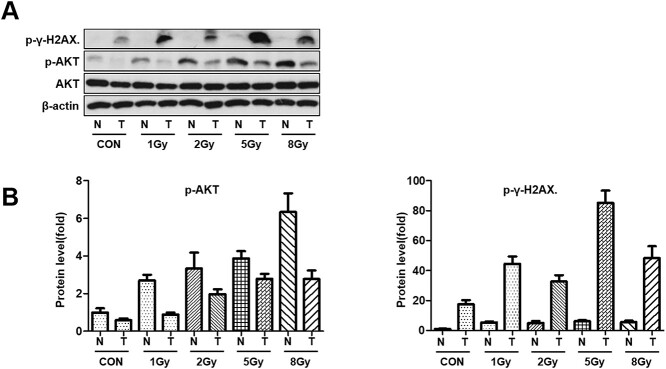
Radioresistance in lung cancer cells is increased by activation of the PI3K/AKT signaling pathway. (A) Expression of phospho-AKT(ser473) and phospho-γ-H2AX(ser139) I at the protein level and (B) Quantification of phospho-AKT and phospho-γ-H2AX protein expression in normal lung tissues and LLC1 tumor tissues after irradiation. N = normal, T = Tumor.

To prove that the PI3K/AKT signaling pathway is activated upon irradiation of NSCLC tumor, we analyzed AKT activation at the protein level using Western blot. LLC1 cell xenografts in the mouse lungs were irradiated with doses ranging from 1 Gy to 8 Gy. After 24 h after irradiation, normal lung tissue and LLC1 tumors were analyzed by probing with anti-phospho-AKT and phospho-γ-H2A.X antibodies. (Fig. 1a and b). It was confirmed that the AKT activation was increased in normal lung and LLC1 tumor tissues upon radiation; however, the expression of phospho-γ-H2A.X, a biomarker for radiation-induced DSBs, was observed to decrease above 5 Gy.

### Effect of irradiation on the colony formation and DNA repair in the NSCLC cells

**Fig. 2:**
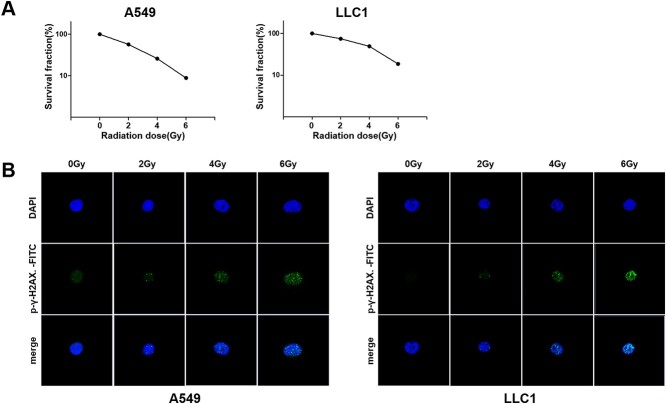
Effect of irradiation on lung carcinoma cells. (A) Dose-survival curves derived from clonogenic assays (A549 *P* < 0.0001, LLC1 *P* < 0.0001). (B) Representative immunofluorescence images of the γ-H2AX expression in A549 and LLC1 cells 6 h after irradiation.

We next performed a colony formation assay to confirm the radiosensitivity of the NSCLC cell lines (Fig. 2a). A549 and LLC1 cells were irradiated with 2 Gy, 4 Gy and 6 Gy and incubated in the complete media for 10–14 days. Decreased survival rates were observed in irradiated A549 and LLC1 cells. Moreover, to confirm the ability to repair DNA DSBs in these cells, the expression of phospho-γ-H2A.X was measured by immunofluorescence (Fig. 2b). The phosphorylation of γ-H2A.X was quantified by confocal microscopy 6 h after irradiation with 2 Gy, 4 Gy and 6 Gy. DSBs in LLC1 and A549 cell lines showed the most increase after 6 h of irradiation with 6 Gy.

### Effect of PI3K isoform-selective inhibitors on PI3K/AKT signaling pathway and cell viability

**Fig. 3:**
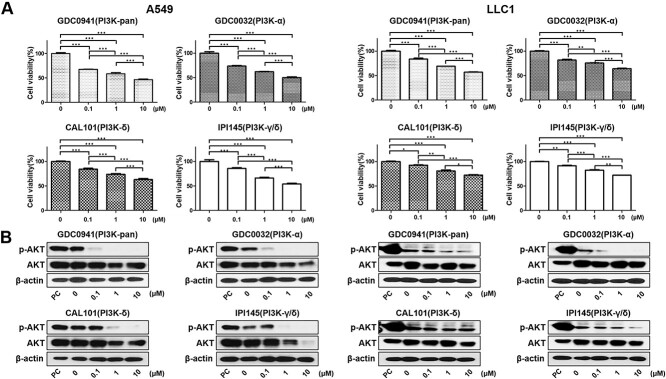
PI3K isoform-selective inhibitors are effective in inhibiting lung carcinoma cell growth and AKT activation. A549 and LLC1 cells were treated with different concentrations of PI3K isoform-selective inhibitors for 24 h (ranging from 0.1 to 10 μM). (A) MTT cell proliferation assay. (B) Representative Western blots of PI3K/AKT signaling activation in A549 and LLC1 cells treated with PI3K isoform-selective inhibitors for 24 h. PC = Positive Control. A *P*-value <0.05 was considered as statistically significant. ^*^P < 0.05, ^*^^*^P < 0.01 and ^*^^*^^*^P < 0.001

To assess the effects of PI3K isoform-selective inhibitors on the viability in NSCLC cell lines, we performed an MTT assay (Fig. 3a). A549 and LLC1 cells were incubated with four different PI3K isoform-selective inhibitors with doses ranging from 0.1 μM to 10 μM for 24 h. The rates of cell viability in A549 cells reduced significantly at higher doses of GDC0941, GDC0032, CAL101 and IPI145 (all *P* < 0.001). Similar effect was observed in the LLC1 cells (*P* < 0.001 with GDC0941, GDC0032, CAL101, except for CAL101 with *P* = 0.139). The mean rates of cell proliferation in A549 cells with four different PI3K inhibitors (PI3K pan-inhibitor GDC0941, PI3K α-isoform inhibitor GDC0032, PI3K δ-isoform inhibitor CAL101 and PI3K γ/δ-isoform inhibitor IPI145) were as follows: 67.54, 72.90, 82.93, 85.03 at 0.1 μM, 58.82, 63.72, 74.39, 65.67 at 1 μM, 45,65, 48.48, 59.62, 54.45 at 10 μM, respectively. The mean rates of cell growth in LLC1 cells with four different PI3K inhibitors were as follows: 83.85, 81.70, 92.60, 91.57 at 0.1 μM, 69.12, 75.52, 80.81, 82.22 at 1 μM, 56.89, 64.18, 51.33, 72.06 at 10 μM, respectively. Furthermore, PI3K inhibitors reduced phosphorylation of AKT in the A549 and LLC1 cells (Fig. 3b). GDC0032 was as effective as GDC0941 in reducing cell viability and activation of AKT. Based on these results, a PI3K α-isoform inhibitor was selected for further experiments, and the therapeutic dose to test the combined effect was determined to be the concentration that reduced the phosphorylation of AKT by approximately 70%.

### Radiation in combination with PI3K isoform-selective inhibitors increases radiosensitivity

**Fig. 4:**
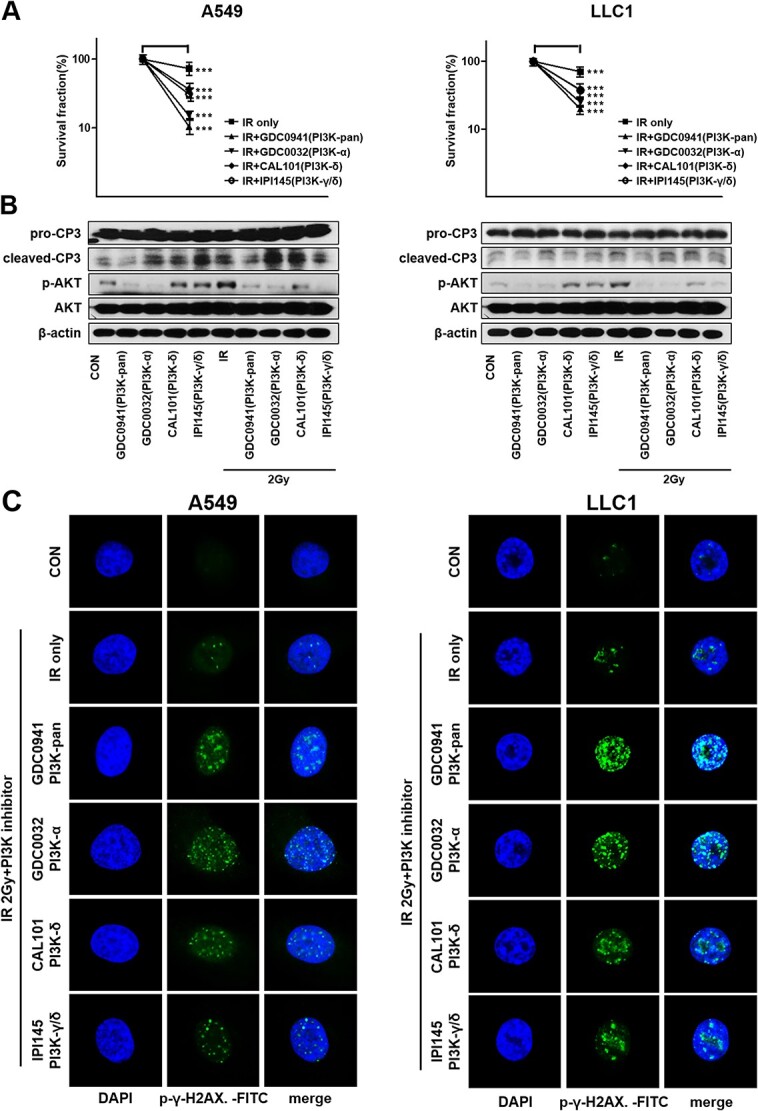
Combining radiation therapy with PI3K isoform-selective inhibitors increases radiosensitivity. (A) Results of clonogenic assays. A549 and LLC1 were treated with PI3K isoform-selective inhibitors (GDC0941 0.1 μM, GDC0032 0.1 μM, CAL101 1 μM, IPI145 1 μM) 3 h after irradiation of 2 Gy. (B) Activation of PI3K/AKT signaling pathway in A549 and LLC1 cells. (C) Representative immunofluorescence images of the γ-H2AX expression in A549 and LLC1 cells after the combination therapy. A *P*-value <0.05 was considered as statistically significant. ^*^P < 0.05, ^*^^*^P < 0.01 and ^*^^*^^*^P < 0.001

To examine the effects of combining radiation and PI3K-isoform inhibitors on radiosensitivity, A549 and LLC1 cells were grown to form colonies for 9–11 days after treating with the combination of radiation and PI3K-isoform inhibitors. Survival curves indicated that a combination of radiation with PI3K isoforms inhibitors suppressed the colony formation in the two cell lines (Fig. 4a). When irradiated with PI3K isoform inhibitors in A549, each survival rate was 20% (PI3K-pan, *P* < 0.001), 25% (PI3K-α, *P* < 0.001), 38% (PI3K-δ, *P* < 0.001) and 37% (PI3K-γ/δ, *P* < 0.001), respectively. The rates were 10% (PI3K-pan, *P* < 0.001), 14% (PI3K-α, *P* < 0.001), 36% (PI3K-δ, *P* < 0.001) and 31% (PI3K-γ/δ, *P* < 0.001) in LLC1 cell line. Although combination of radiation with PI3K- δ isoform inhibitor (CAL101) and PI3K- γ/δ (IPI145) inhibitor also showed decreased survival rates, the PI3K α-isoform inhibitor (GDC0032) inhibited colony formation the most. Its inhibition rate was comparable to that of the PI3K pan-inhibitor (GDC0941). Also, the expression of cleaved-caspase 3, an important marker of cell apoptosis, was increased in combination therapy. In particular, the expression of cleaved-caspase 3 increased in PI3K- α isoform inhibition as much as in PI3K-pan inhibition. The AKT activation increased by irradiation was decreased by PI3K-isoform inhibitors (Fig 4b). To assess the effects of combination treatment on DNA repair, we measured phospho-γ-H2A.X by immunofluorescence staining 6 h after the combination therapy (Fig 4c). The expression of phospho-H2A.X was increased by the combination of radiation and PI3K- isoform inhibitors. In particular, the inhibition of DNA DSB induced by the PI3K-α isoform inhibition was comparable to that observed with PI3K-pan inhibition.

### Effects of PI3K-isoform inhibition of cell migration, PI3K/AKT pathway activation and expression of EMT-associated genes by in NSCLC cell lines

**Fig. 5:**
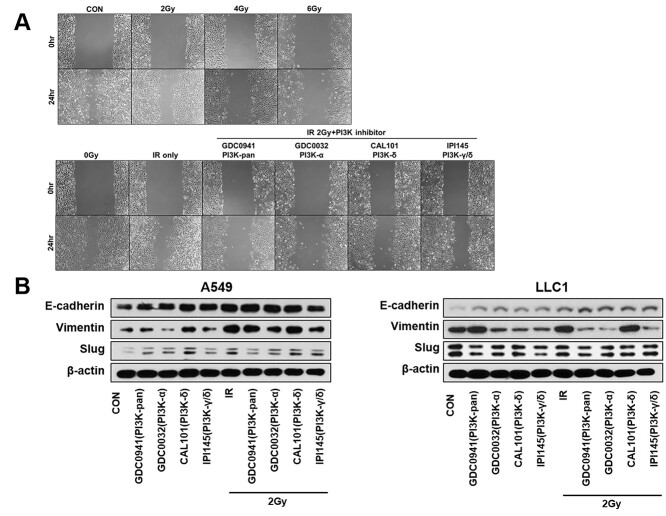
Combining radiation with PI3K isoform-selective inhibitors regulates EMT-associated signaling in NSCLC cell lines. (A) The ability of migration and (B) expression of EMT markers in treated and untreated A549 and LLC1 cells with PI3K isoform-selective inhibitors 24 h after irradiation. IR = irradiation.

We performed a wound healing assay to determine the effects of PI3K-isoform inhibition in combination with irradiation on the migration ability of cancer cells (Fig. 5a). Combination PI3K-α-isoform inhibition and radiation significantly decreased the migration ability of A549 cells as compared to that of the cells in the radiation-only group. EMT is considered a major reversible step that promotes tumor metastasis. We measured the expression of EMT-associated signaling molecules in the cells treated with a combination of radiation and the inhibitors (Fig. 5b). We probed E-cadherin as an epithelial marker, vimentin as a mesenchymal marker, and Slug as an EMT inducer. Notably, PI3K-isoforms inhibition increased the expression of E-cadherin and decreased the expression of Slug as compared to those in the control group. In addition, PI3K-isoforms inhibition significantly decreased vimentin expression in LLC1 cells, and marginally it decreased in A549 cells (Supplementary Fig. 1).

### Inhibition of PI3K-α isoform enhances NSCLC tumor radiosensitivity *in vivo*

**Fig. 6:**
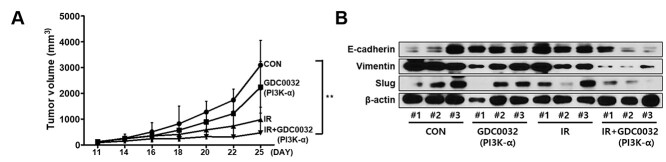
Combining radiation with the PI3K-α isoform inhibitor suppressed lung tumor growth. After subcutaneous injection of LLC1 cells, irradiation (5 Gy) with or without treatment of the GDC0032 (5 mg/Kg body weight) was carried out. (A) Tumor volume growth was measured from 1–25 days. (B) Expression of EMT markers in the lung tumor tissue. IR = irradiation. A *P*-value <0.05 was considered as statistically significant. ^*^P < 0.05, ^*^^*^P < 0.01 and ^*^^*^^*^P < 0.001.

To confirm the effect of inhibition of PI3K-α signaling in NSCLC tumors, we xenografted LLC1 cells subcutaneously into C57BL/6 mice. The xenograft tumors were irradiated with 5 Gy of 1 fraction with or without 5 mg/kg PI3K-α inhibitor(GDC0032) treatment. The combination of radiation with PI3K-α inhibitor improved NSCLC radiosensitivity and markedly delayed tumor growth *in vivo* than in the radiation-only group (*P* < 0.05) (Fig. 6a). We also observed the changes in EMT-associated signaling (E-cadherin, vimentin and Slug) in the tumor tissue treated with PI3K-α inhibitor, where the EMT signaling was inhibited significantly (Fig. 6b). Moreover, these data suggested that inhibition of PI3K-α isoform was sufficient to sensitize tumors to radiation.

## DISCUSSION

Radioresistance is one of the major causes of failure in cancer therapy. PI3K pathway has been proven to play a crucial role in the progression of and radioresistance in several types of cancer [6, 21]. Many studies have shown that activation of AKT, which is an important component of the PI3K/AKT signaling pathway, is associated with radioresistance in malignant tumors [14, 22, 23]. Therefore, the inhibition of AKT activation combined with radiation could be effective in overcoming radioresistance and increasing radiosensitivity. One of the other major reasons is that the activation of AKT promotes the survival of cancer cells exposed to irradiation by inhibiting apoptosis [24, 25]. Additionally, several researchers have reported that repair of radiation-induced DSBs and radioresistance depend in part on activation of the AKT *in vitro* and *in vivo* [9, 26–29].

DSBs are the major cause of irradiation-induced cell death. DSBs are rarely induced endogenously and are primarily radiation-induced. DSBs start to appear at a dose of a few mGy and increase as the radiation dose increases. Radiation induces simple and complex DSBs with 3′ blocked ends [30–32]. DSB repair is regulated by the BRCA1 gene, which is activated by AKT [33]. Considering the role of the PI3K/AKT pathway in DSB repair, inhibition of AKT activation during irradiation can prevent DSB from being repaired [5, 34, 35]. In several studies on lung cancer, it has been reported that AKT activity affects radiation-induced DSB repair and significantly changes survival rates [5, 9, 35]. Furthermore, some researchers have demonstrated improved radiosensitivity by inhibiting the AKT activation and several other genes [36–39]. In our study, levels of AKT and γ-H2A.X phosphorylation in LLC1 tumor tissues and normal lung tissues were analyzed *in vivo* for the first time. The excessive activation of AKT reduced the expression of phospho-γ-H2A.X, which indicated the induction of radioresistance. Our results were consistent with other studies that reported that the activation of AKT may reduce the efficacy of radiotherapy by promoting DNA repair in NSCLCs [35, 40].

PI3K inhibitors might serve as a potential remedy for radioresistance. However, in the relevant clinical trials, it has been reported that first-generation pan-PI3K inhibitors have limited efficacy and relatively high rates of side effects [41]. Because, after irradiation, AKT is more activated in normal cells than in tumor cells, as shown in our study as well [42, 43], PI3K-inhibitor may exert potent effect on normal cells than on tumor cells. Thus, selective therapeutic effects with reduced side effects can be expected after using PI3K-isoform inhibitors. Moreover, in our study, it was established that the PI3K-α inhibitor did not significantly affect the expression of phospho-AKT in normal tissues *in vivo* and no weight loss or disease was seen in mice. PI3K has four isoforms of the catalytic subunit: p110α, p110β, p110δ and p110γ [44, 45]. The four p110 class I PI3K isoforms have specific functions in different types of tumors [4, 45]. Most of the p110α and p110β isoforms are expressed in solid tissues, whereas most of the p110δ and p110γ isoforms are expressed in the cells of a hematopoietic lineage [46]. It has been well established that p110α is overexpressed in human cancers and has an oncogenic potential [47, 48]. p110β plays a role in stimulating cell proliferation and invasive cell growth [49]. The role of the δ isoform has also been explored in several studies. It is important for the activation of AKT in acute myeloid leukemia and cell migration and chemotaxis in tumor progression of breast cancer [50, 51]. Recent papers have reported that the γ isoform is involved in tumor angiogenesis and drug resistance of chronic myeloid leukemia cells [52, 53].

However, the role of the PI3K isoforms in radioresistance in NSCLCs remained unclear. In this context, we explored the role of the PI3K inhibitors in irradiated NSCLC cells and tried to find the most effective isoform-selective inhibitor. In addition, we selected a concentration that showed 70% AKT activity inhibition for a particular dose of PI3K-isoform inhibitors to minimize side effects. We found that the PI3K signaling pathway modulates the radiosensitivity of NSCLC cell lines. Therefore, irradiation with PI3K isoform-selective inhibitors could delay the DSB repairs and increase radiosensitivity. In particular, PI3K-α inhibitor was as effective as PI3K-pan inhibitors, which implies that PI3K-α-isoform might play a key role in the radioresistance of NSCLC cells. Moreover, the expression level of the activated caspase-3, which indicates the occurrence of radiation-induced apoptosis, was elevated after the inhibition of PI3K-α than after the inhibition of other isoforms [54]. Our results were consistent with previous studies on PI3K-α inhibitor in other cancers [55–57]. Another interesting finding from our experiments involving LLC1 xenografts mouse model was that no weight loss or illness was observed during the experimental period in any of the mice, despite the fact that the concentrations of PI3K- α inhibitors used in our study were similar to those used previously [58–60]. This result suggests that the PI3K-α inhibitor may be the most effective agent with promising safety. However, we believe that further studies are needed to determine the effects of PI3K-α inhibitors on NSCLC.

The PI3K pathway plays an important role in EMT, and its activation was found to be increased in several invasive cancer cells [4, 26, 27, 61–63]. Recently, many researchers have become interested in the relationship between radiation and EMT. Several papers have demonstrated that irradiation can promote EMT in several types of cancer [64–67]. EMT was observed to increase in tumors that acquired resistance to radiation and anti-cancer therapy [54, 68, 69]. In our study, we observed that PI3K-α-isoform had more relevance than the other isoforms in EMT related to radioresistance. We analyzed the changes in the expression level of EMT markers such as E-cadherin, vimentin and Slug *in vitro* and *in vivo.* E-cadherin and Slug are associated with EMT and are known to be involved in modulating radioresistance [69]. The expression of Slug was associated with EMT and resistance to anti-cancer therapy in lung cancer [70]. It was also reported that increased expression levels of Slug by downregulation of E-cadherin promoted metastasis in lung cancers [63, 71]. Vimentin is a direct substrate of AKT, the immediate downstream effector of PI3K, and this may constitute a mechanism by which the PI3K pathway contributes to EMT [72]. In our study, a combination of irradiation and PI3K isoform inhibition increased the expression of E-cadherin and decreased the expression of vimentin and Slug, suggesting a decrease in radioresistance (Fig. 5b). In particular, the *in vitro* and *in vivo* modulation in the expression of EMT markers in LLC1 cells induced by the combination treatment of irradiation and PI3K-α-isoform inhibitor was comparable to that by the treatment with the PI3K-pan isoform inhibitor. When the A549 cells were treated only with the PI3K inhibitors, the expression of Slug was increased; however, its expression was decreased when the cells were treated with radiation as well. Additionally, in the wound healing assay, the reduction in the cell migration by the combination of PI3K-α isoform inhibition and radiation was comparable to that by the PI3K-pan inhibition. It suggested that the irradiation combined with the inhibition of PI3K-α isoform increases radiosensitivity and reduces metastatic capacity.

Buparlisib (BKM120) is an oral pan class 1 PI3K inhibitor, which has been studied in preclinical and clinical studies. After establishing favorable pharmacokinetics with acceptable toxicity, the first phase I clinical trial of Buparlisib with palliative thoracic radiotherapy in NSCLC patients was conducted [73, 74]. In particular, 67% of the patients exhibited 20% median reduction in tumor hypoxic volume. This trial showed that PI3K inhibition reduces tumor hypoxia in NSCLC patients and is well tolerated when combined with thoracic radiotherapy. It supported the development of other clinical trials combing these types of agents and radiotherapy. The finding that most glioblastoma tumors exhibit activation of the PI3K pathway led to the studies of Buparlisib in glioblastoma. A phase I, two-stage, multicenter, dose-escalation study of Buparlisib in combination with temozolomide and radiotherapy was performed in patients with newly diagnosed glioblastoma [75]. However, the maximum tolerated dose could not be established because of the challenging safety profile and high rate of treatment discontinuation due to adverse events. Currently, among several ongoing clinical trials of Buparlisib in head and neck cancers, only one involves combination with radiotherapy (NCT02113878). Paxalisib (GDC-0084) has been investigated in cancers, especially in brain tumors. An ongoing phase 1 study is testing concurrent treatment of GDC-0084 with radiation in the patients with solid tumor brain metastases or leptomeningeal metastases harboring PI3K pathway mutations (NCT04192981). A phase I study involving GDC-0084 treatment after radiotherapy in pediatric patients with newly diagnosed diffuse midline glioma is also underway (NCT03696355). However, clinical studies testing the combination of isoform-selective PI3K inhibitors and radiotherapy are still lacking.

Our study has several limitations. First, we could not verify the inhibitory effect of other isoform-inhibitions on the *in vivo* tumor growth. Second, the reason why PI3K-α-isoform inhibition regulates EMT and radioresistance more effectively than the inhibition of other isoforms of PI3K is not explained in this study. Further studies elucidating the downstream factors are warranted to validate our findings. Nevertheless, our study found that inhibition of PI3K-α isoform influences radiosensitivity in NSCLCs and confirmed that it also suppresses EMT.

In conclusion, the combination of radiation with PI3K-α-specific inhibition shows antitumor efficacy in NSCLC by modulating tumor growth, cell survival and EMT. We propose that the PI3K-α isoform inhibition is a potentially effective strategy to overcome radioresistance safely and efficiently. In the future, relevant clinical studies are required to help understand the mechanism and clarify the actual benefit to cancer patients.

## FUNDING

This study was supported by a Dongin Sports research grant of Yonsei University College of Medicine (6–2017-0105).

## DATA AVAILABILITY

Research data are stored in an institutional repository and will be shared upon request to the corresponding author.

## CONFLICT OF INTEREST

None of the authors have any conflicts of interest to disclose.

## Supplementary Material

Supplement_data_rrac018
