# Supplementary material for: Combining radiation with PI3K isoform-selective inhibitor administration increases radiosensitivity and suppresses tumor growth in non-small cell lung cancer
Source: J Radiat Res. 2022 May 9;63(4):591–601. doi: 10.1093/jrr/rrac018 (PMC9303607; doi:10.1093/jrr/rrac018)
Supplement: Supplement_data_rrac018 [file supplement_data_rrac018.docx]

**Supplement Table 1. Information of the antibodies used**

|  | **Antibody** | **Cat. No.** | **Brand** |  |
| --- | --- | --- | --- | --- |
|  | AKT | 9272 | Cell signaling, Massachusetts, USA |  |
|  | Phospho-AKT | 9271 | Cell signaling, Massachusetts, USA |  |
|  | Caspase 3 | 9665 | Cell signaling, Massachusetts, USA |  |
|  | E-cadherin | 610181 | BD biosciences, san Jose ca, USA |  |
|  | Vimentin | 5741 | Cell signaling, Massachusetts, USA |  |
|  | Slug | 9585 | Cell signaling, Massachusetts, USA |  |
|  | β-actin | sc-47778 | Santa cruz, Dallas, Texas, USA |  |

**Supplement Fig. 1 Expression of vimentin in A549 cells.**

Expression of vimentin in treated and untreated A549 cells with PI3K isoform–selective inhibitors 24 h after irradiation.

**
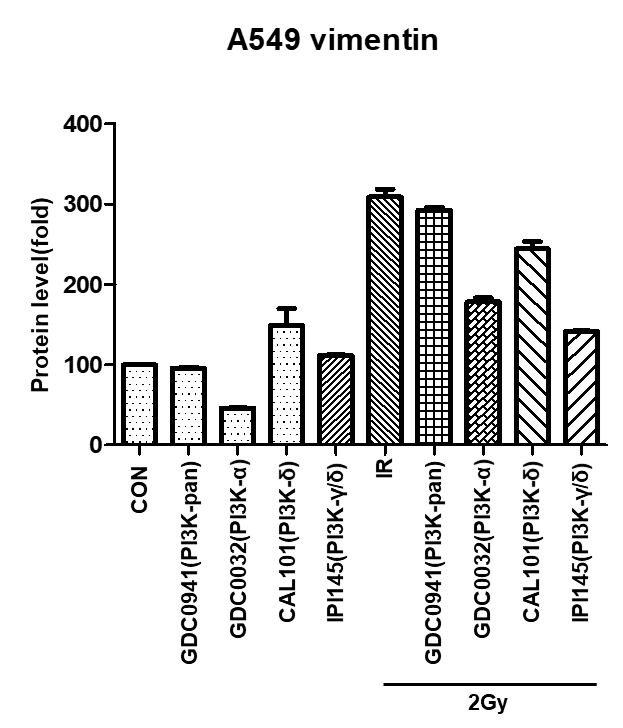
**
